# Premature ovarian insufficiency as a variable feature of blepharophimosis, ptosis, and epicanthus inversus syndrome associated with c.223C > T p.(Leu75Phe) *FOXL2* mutation: a case report

**DOI:** 10.1186/s12881-019-0865-0

**Published:** 2019-07-31

**Authors:** Barbara Grzechocińska, Damian Warzecha, Maria Wypchło, Rafal Ploski, Mirosław Wielgoś

**Affiliations:** 1 1st Department of Obstetrics and Gynecology, Medical University of Warsaw, Pl. Starynkiewicza 1/3, 02-015 Warsaw, Poland; 2 Department of Medical Genetics, Medical University of Warsaw, ul Pawinskiego 3c, 02-106 Warsaw, Poland; 3 Postgraduate School of Molecular Medicine, Medical University of Warsaw, Warsaw, Poland

**Keywords:** Blepharophimosis, ptosis, and epicanthus inversus *s*yndrome (BPES), Premature ovarian insufficiency (POI), Genetics, Amenorrhea, Case report

## Abstract

**Background:**

*FOXL2* gene mutations cause blepharophimosis-ptosis-epicanthus inversus syndrome (BPES) and may be associated with premature ovarian insufficiency (POI). Two types of BPES were described in the literature. BPES type 2 is a simple association of inherited developmental defects of the eyelid area, while in type 1 female patients additionally suffer from POI. The following case study is the first report of endocrine impairments typical for menopausal transition in young female with NG_012454.1:g.138665342G > A, c.223C > T p.(Leu75Phe), mutation in *FOXL2* gene. This mutation has been reported in the literature before, however until now, it was never linked to BPES type 1.

**Case presentation:**

An 18-year-old nulliparous woman suspected of secondary amenorrhea was referred to our Endocrinology Outpatient Clinic. Blood tests revealed decreased levels of AMH (anti-Mullerian hormone) and increased levels of gonadotropins, suggesting menopausal transition. Her past medical history was remarkable for several ophthalmic defects that has required surgical interventions. BPES syndrome had not been suspected before, although the patient had reported a similar phenotype occurring in her father, sister and half-sister. Venous blood samples were collected from the female proband and from her three family members. Whole-exome sequencing and deep amplicon sequencing were performed. A potential pathogenic variant in the *FOXL2* gene was revealed. Namely, the c.223C > T p.(Leu75Phe) missense variant was detected.

**Conclusions:**

The authors found mutations, c.223C > T p.(Leu75Phe) in the *FOXL2* gene in a young woman with hormonal disorders suggesting menopausal transition. These results indicate that the possibility of different phenotypes should be considered in patients with a similar genetic mutation.

## Background

The term blepharophimosis, ptosis, and epicanthus inversus *s*yndrome (BPES) derives from the association of several clinical features in those patients affected with the syndrome. The prevalence of the syndrome in the general population is unknown but is estimated at 1 per 50,000 people. Pedigree analyses in affected families have indicated an autosomal dominant pattern of inheritance [1, 2]. In 1983, Zlotogora et al. first described two phenotypes of BPES, with the differentiating factor being the concomitant presence of premature ovarian insufficiency (POI) [3]. BPES type 2 is the simple association of inherited developmental defects of the eyelid area, while in BPES type 1, female patients suffer in addition from POI.

All the BPES cases described in the literature are caused by inherited or *de-novo* mutations of the *FOXL2* gene (chr. 3q23) or of its regulatory regions [4]. The structure of *FOXL2* contains two domains: the DNA-binding forkhead domain and the 14-residue polyAla tract. The *FOXL2* gene encodes a transcription factor involved in the development of eyelids and ovarian follicles and it is selectively expressed in mesenchymal tissue [5]. The majority of patients affected by BPES have no other inherited defects, although several cases of coexisting mental retardation were described in cases where there were large deletions of chromosome 3 [6, 7].

POI is diagnosed when the cessation of menses occurs, due to ovarian failure, before the age of 40 years [8]. POI affects up to 1% of women in the general population. The background of this condition may be iatrogenic (oophorectomy, aggressive chemotherapy, or radiotherapy), autoimmune, or genetic (Turner or Fragile X syndrome, or BPES). The prevalence of chromosomal abnormalities in patients with POI was estimated at 12.1% [9].

The clinical findings for patients who undergo natural menopause are similar to those with POI and include: hypergonadotropic hypogonadism and involutional changes in the reproductive system. There are several tests which evaluate the patient’s remaining reproductive potential and may predict forthcoming menopause [10, 11]. Among women of reproductive age, elevated FSH levels (> 20 IU/L), and very low estradiol indicate the possibility of a POI diagnosis. A low atrial follicles count (AFC < 4) is also useful in evaluating the remaining ovarian reserve. These results suggest that problems in becoming pregnant are possible, even after IVF [12]. Furthermore, POI significantly increases the risk of cardiovascular disease, osteoporosis, and mood or cognitive impairments [13].

The following study is a case report of a Polish family diagnosed with blepharophimosis, ptosis, and epicanthus inversus *s*yndrome type 1 associated with c.223C > T p.(Leu75Phe) mutation in the *FOXL2* gene. The paper presented here is a retrospective analysis of the diagnostic process and the clinical findings, therefore ethics committee approval was not required. All participants described below have given their written informed consent to the publication of these findings. On behalf of the minor half-sister of the proband (II:4), her parents provided written consent for the genetic testing an publication of obtained results.

## Case presentation

**Fig. 1:**
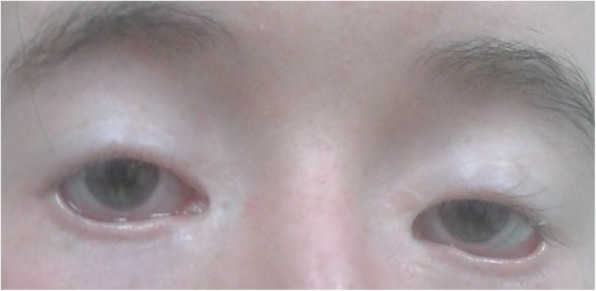
Ophthalmological phenotype of the study patient

An 18-year-old nulliparous woman (II:3) was referred to the Endocrinology Outpatient Clinic of the 1st Department of Obstetrics and Gynecology at the Medical University of Warsaw, due to secondary amenorrhea. The patient’s most recent menstrual period had occurred 6 months prior. On admission, the patient was in a generally good condition, without any other remarkable symptoms, and with BMI 18.5 kg/m2 (174 cm tall and 56 kg weight), and Tanner stage V (breast and pubic hair). Menarche occurred at 13y. Previously to the amenorrhea, the patient had reported irregular menstrual cycles. Upon physical examination, acne and abnormal eyelid development with ptosis and microphthalmia (a corneal diameter of less than 10 mm and an antero-posterior diameter of the globe of less than 20 mm), were observed. Her past medical history was remarkable for several ophthalmic defects that had required surgical interventions. At 1 year old, the patient had received frontalis suspension surgery using an autogenous graft (fascia lata) to treat blepharoptosis and poor levator muscle function. A year later, this was followed by lateral canthopexy. Finally, in 2015, II:3 underwent canthoplasty to correct the epicanthus inversus, and telecanthus; however, BPES syndrome was not suspected at that time, despite the patient reporting a similar phenotype (concerning ophthalmic alternations, Fig. 1) occurring in her father (I:1, Fig. 2), sister (II:1) and half-sister (II:4).

**Fig. 2:**
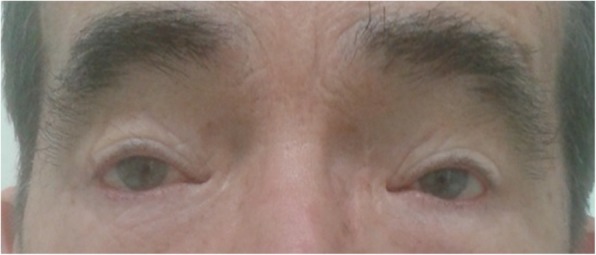
Ophthalmological phenotype of patient’s father (I:1)

Hormonal diagnosis was performed. The progesterone withdrawal test was positive. Serum hormone levels were measured on the 3rd day of the menstrual cycle. Due to low estradiol levels, hormonal replacement therapy was introduced (estradiol 2 mg daily between the 5th and 26th days of the menstrual cycle, and dydrogesterone 10 mg daily between the 15th and 26th days). Blood samples and laboratory tests were performed every 3 months. The results of the blood tests are presented in Table 1. Initially, elevated levels of FSH (21.9 [IU/L]), normal LH and decreased levels of estradiol (5.3 [pg/ml]) were detected. During the follow-up period these levels tended to normalize; however Anti-Müllerian hormone (AMH) concentrations decreased to below the critical threshold (0.52 ng/ml). These data are presented in Table 1.

**Table 1:** Hormone profiles during observation

Hormone	Before	After 3 months	After 6 months	After 1 year
FSH [mIU/ml]	21.9	19.1	14.1	8.8
LH [mIU/ml]	9.3	15.4	12.4	15.3
Estradiol [pg/ml]	5.3	22.66	23.67	205.5
Prolactine [ng/ml]	33.1	36.0	3.3	15.3
AMH [ng/ml]	1.29	N/A	0.93	0.52
Testosterone [ng/ml]	0.55	0.36	0.4	N/A
Glucose [mg/dl]	104	N/A	N/A	N/A
Insulin [mU/l]	13.8	N/A	N/A	N/A
HOMA-IR	3.5	N/A	N/A	N/A

*FSH* Follicle-stimulating hormone, *LH* Luteinizing hormone, *AMH* anti-Mullerian hormone, *DHEA-S* Dehydroepiandrosterone sulfate, *HOMA-IR* Homeostatic model assessment-insulin resistance

Adrenal hormones were also measured during endocrine investigation. Elevated 17-OHP levels were observed. To exclude primary adrenal insufficiency the authors performed conventional-dose short test with 250 μg of synthetic adrenocorticotropic hormone (Synacthen test) (Table 2). This test revealed appropriate adrenal function and the patient was scheduled for a follow-up observation.

**Table 2:** The results of Synacthen test (intravenous, conventional-dose short test with 250 μg of synthetic adrenocorticotropic hormone) in patient II:3

Hormone	Serum concentration before test	30 min after Synacthen injection	60′ minutes after Synacthen injection
17-OHP [ng/ml]	3.87	5.43	7.08
Testosterone [ng/ml]	0.55	0.48	0.5

The patient’s pelvic ultrasound results remained unremarkable (normal uterus and ovaries). The antral follicle count (AFC) was in the normal range, with 5 follicles in the right ovary and 9 in the left.

The other three family members (I:1, II:1 and II:4) with suspected BPES were also examined. In all of them signs of blepharophimosis, ptosis, and epicanthus inversus were identified. No remarkable medical history was obtained. II:1 (34y) at the age of 14 underwent menarche and up to that age had had regular menses; while II:4 (14y) had not yet reached menarche. In this latter case, due to the suspected genetic background of those anomalies and a phenotype suggesting BPES, the patient was referred to a geneticist.

## Materials and methods

Venous blood samples were collected from the proband and her three family members (father, sister and half-sister). DNA was extracted using the standard salting-out method. Written informed consent was obtained from all subjects prior to genetic testing.

### Whole exome sequencing (WES)

WES was performed on the father’s sample using SeqCap EZ MedExome (Roche, Basel Switzerland) on HiSeq 1500 (Illumina, San Diego, CA). The bioinformatic analysis of the WES data was performed using the previously described pipeline. The mean depth of coverage in the sequenced sample was 93x, with a 20x coverage for 97% of the target sequence and a minimum of 10x coverage for 99%. In order to confirm the WES results and to verify the carrier status of the participants of the study, Amplicon Deep Sequencing (ADS) was performed using Nextera XT Library Preparation Kit (Illumina, San Diego, CA). Sequencing was carried out on the HiSeq 1500 system. The following PCR primers were used for ADS: 5′- aca gtc aag gag cca gaa gg - 3′, and 5′- ggt cca gcg tcc agt agt tg − 3′.

**Fig. 3:**
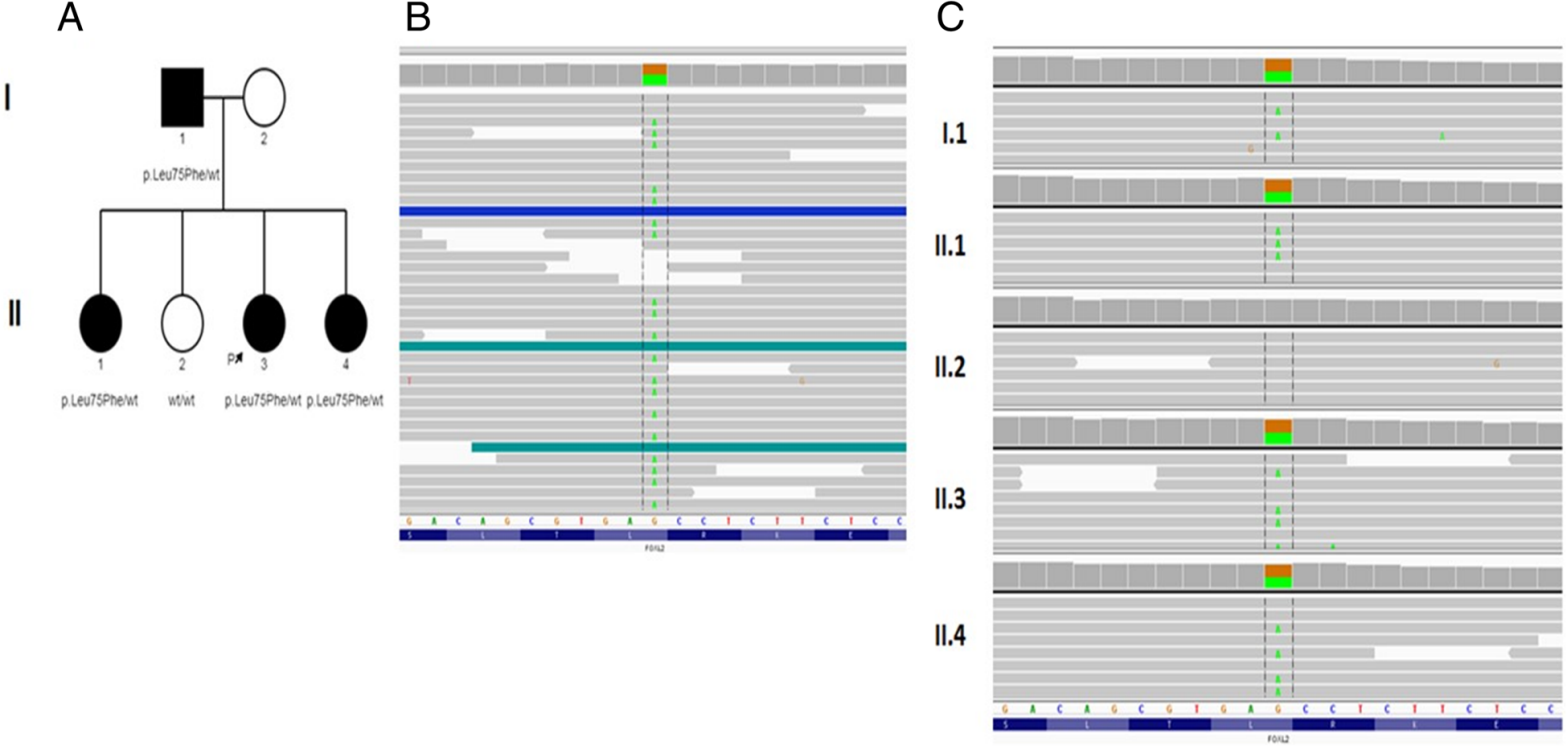
Sequencing results: **a** Pedigree of studied family, proband is marked with black arrow. **b** Next-generation Sequencing results of c.223C > T p.(Leu75Phe) variant in the *FOXL2* gene in the proband using Integrative Genomic Viewer (IGV). **c** Amplicon deep sequencing (ADS) results in the *FOXL2* gene in the studied family (IGV view)

The bioinformatic analysis of the WES data revealed one potential pathogenic variant in the *FOXL2* gene (Table 3). Analysis of the family’s samples using amplicon deep sequencing (ADS) confirmed the presence of the heterozygous variant in the *FOXL2* gene in the proband, father, and two affected daughters (Fig. 3).

**Table 3:** *FOXL2* variant in proband

cDNA level	Protein	Gene	Reference transcript	Chromosomal position (hg19)	Zygosity
c.223C > T	p.(Leu75Phe)	*FOXL2*	NM_023067.3	chr3:138665342-G > A	heterozygous

The CADD (combined annotation dependent depletion) score indicating the likely deleteriousness of the c.223C > T p.(Leu75Phe) missense variant allele was 22.2. Moreover, the pathogenicity estimation by the Polyphen2 and Mutation Taster also predicted this mutation’s deleteriousness. The c.223C > T p.(Leu75Phe) variant has neither been reported in the GnomAD (http://gnomad.broadinstitute.org) database, nor in our own database which includes more than 1000 WES samples taken from the Polish population. In the proband no plausible rare variants (frequency < 0.0001 in both mentioned databases) in other genes were found. FASTQ and VCF files from WES are available on request to qualified researchers.

## Discussion and conclusions

The present study is the first published report on BPES in the Polish population. PBES is usually suspected based on the patient’s phenotypic characteristics. Although our proband displayed ophthalmic signs typical of the syndrome, and had undergone several corrective ophthalmologic surgeries, PBES had not previously been diagnosed.

Several types of rearrangements in the 3q23 region affecting the *FOXL2* gene have been described in the literature. It has been suggested that *FOXL2* mutations truncating the protein before polyalanine tract are associated with POI, while those causing polyalanine expansion tend to lead to BPES type 2 [14, 15]. However, in cases where other mutations are described (such as missense mutations or microdeletions), the genotype-phenotype correlation was not apparent [14, 16].

The c.223C > T p.(Leu75Phe) mutation in the *FOXL2* gene was previously found by Xue et al. in a study of three women from two Chinese families [17]. In that study, two women in family A and one woman in family B had typical eyelid malformations, which were the same as those found in our patient and her affected family members. The first of the Chinese subjects was 63 years old, following menopausal onset at age 52y. The second and the third subjects were of reproductive age and with regular menstrual cycles. None of the subjects had fertility problems. However, none of those subject’s hormone status is known as the investigators did not carry out any hormonal tests. Careful interpretation of the genetic tests, in order to predict the risk of POI, is advisable [14].

Hormonal results in our study suggest there is a correlation between the type 1 specific genotype and hormonal disturbances. Although they are not typical, they may still indicate a poor ovarian reserve.

There are several tests which evaluate a subject’s remaining reproductive potential and which may be early signs of approaching menopause [10, 18]. Elevated FSH serum concentrations (21.9 IU/L) and very low estradiol levels (5.3 [pg/ml]) in patients, may point to the possibility of POI. Small atrial follicles (2–8 mm in diameter) are also an indicator of the remaining ovarian reserve. An AFC count of less than 4, suggests the subject will likely have problems with becoming pregnant, even after IVF [11]. In our study, the AFC counts were 5 and 9, in the right and left ovaries, respectively. AMH produced by pre-antral and small antral ovarian follicles are acknowledged markers of the ovarian reserve and the remaining reproductive capacity of the subject. Current guidelines of the American College of Obstetricians and Gynecologists recommend that AMH serum concentrations should be evaluated as one of the best tools for measuring the remaining ovarian reserve [12]. Both AFC counts and AMH concentrations have good predictive value, however the latter is preferred for ovarian reserve measurement, especially if the findings obtained from both tests persist [19, 20]. Our study demonstrated that consistently low AMH serum levels, below 0.52 ng/ml in the proband with the c.223C > T p.(Leu75Phe) mutation, allowed the detection of the subject’s decreased ovarian reserve.

Genetic testing in patients affected with POI is highly recommended, especially if iatrogenic causes are excluded [21]. Molecular tests should also be conducted in relatives, especially in adolescent females and in women at a reproductive age, as early diagnosis helps women retain their future fertility. For the women described in our study, fertility preservation methods (cryopreservation of the oocytes, or ovarian tissue) were offered.

The young ago for the patients and their decreased estradiol levels were indications for replacement therapy. II:3 preferred an oral substitutive hormonal therapy of 2 mg of estradiol daily, and 10 mg of dydrogesterone in the second phase.

Hormone testing of our patient did not unambiguously indicate preterm menopause, and it is difficult to assess whether the patient’s current hormonal disorders are transient or will indeed lead to menopause. The patient remains under constant observation. It is worth emphasizing that this paper is the first published description of the menopausal transition in a patient with the *FOXL2* gene mutation that has been linked to BPES type 2.

We found mutations in the c.223C > T p.(Leu75Phe) *FOXL2* gene in young women with hormonal alternations typical of POI. These results indicate the possibility that different phenotypes should be considered in patients with similar genetic mutations.

## Data Availability

The datasets used and analysed during the current study are available from the corresponding author upon reasonable request.

## Abbreviations

ADS: Amplicon Deep Sequencing
AFC: Atrial follicle count
AMH: Anti-Müllerian hormone
BPES: Blepharophimosis, ptosis, and epicanthus inversus syndrome
POI: Premature ovarian insufficiency
